# Testing Two Student Nurse Stress Instruments in Chinese Nursing Students: A Comparative Study Using Exploratory Factor Analysis

**DOI:** 10.1155/2020/6987198

**Published:** 2020-10-07

**Authors:** Yiru Zhu, Yanjin Liu, Lina Guo, Martyn C. Jones, Yuanli Guo, Suyuan Yv, Yvru Guo, Genoosha Namassevayam, Miao Wei

**Affiliations:** ^1^Department of Neurology, The First Affiliated Hospital of Zhengzhou University, Zhengzhou, 450052 Henan Province, China; ^2^Department of Nursing, The First Affiliated Hospital of Zhengzhou University, Zhengzhou, Henan, China; ^3^School of Nursing and Health Sciences, University of Dundee, 11 Airlie Place, Dundee DD1 4HJ, UK; ^4^Department of Osteology, The First Affiliated Hospital of Zhengzhou University, Zhengzhou, 450052 Henan Province, China; ^5^Department of Supplementary Health Sciences, Faculty of Health-Care Sciences, Eastern University, Sri Lanka

## Abstract

**Background:**

The development and transformation of nursing within professional tertiary education have exerted a great pressure and challenge upon nursing students. Stress experienced by nursing students is a common precursor of psychological distress and attrition. However, no scale is specifically used to evaluate the sources of stress experienced by nursing students in Mainland China. *Aims and Objective*. This study is aimed at testing and comparing the reliability and validity including sensitivity and specificity of two nursing students' stress instruments, the Chinese version of Student Nurse Stress Index Scale (SNSI-CHI), and the Stressors in Student Nursing Scale (SINS-CN) in Chinese nursing students, and describing the stress status of nursing students in China.

**Methods:**

A cross-sectional survey was conducted in two nursing schools in Henan Province from August 2017 to January 2018. Data were collected by using a questionnaire comprising the Chinese version of SNSI (SNSI-CHI), the Chinese version of SINS (SINS-CN), and the Chinese Perceived Stress Scale (CPSS). Homogeneity and stability, content, construct and concurrent validity, and sensitivity and specificity were assessed.

**Results:**

The Cronbach's alpha (*α*) of SNSI-CHI was 0.90, and the item-to-total correlations ranged from 0.35 to 0.66. The Cronbach's *α* of SINS-CN was 0.93, and the item-to-total correlations ranged from 0.19 to 0.61. The findings of exploratory factor analysis (EFA) confirmed a good construct validity of SNSI-CHI and SINS-CN. The Pearson's rank correlation coefficients, between total scores of SNSI-CHI and CPSS and SINS-CN and CPSS, were assessed to 0.38 (*P* < 0.01) and 0.39 (*P* < 0.01), respectively. Regarding the CPSS, as the criterion, the cut-points of SNSI-CHI and SINS-CN for the area under the receiver operator characteristic (ROC) curve were 0.77and 0.66, respectively.

**Conclusion:**

Both scales are valid and reliable for evaluating the source of stress of student nurses in China. Each has its own characteristics, but the SNSI-CHI demonstrated marginal advantage over the SINS-CN. The SNSI-CHI is short, is easily understood, and with clear dimension for the nursing students, and the SNSI-CHI is more acceptable for the users in China.

## 1. Introduction

Stress researcher Selye has defined stress as reaction that produces nonspecific responses in the body when faced with demands for change [1]. Stress is a dynamic interaction between the individual and the environment, with the stress process consisting of antecedents (sources of stress or demand), process (coping), and stress outcome elements (e.g., emotions such as anxiety and distress) [2, 3]. Perceptions of stress occur when there is an imbalance between the demand for resources and an individual's ability to cope [4]. Stress has been identified as a major disease of the Twentieth century [5, 6], which has been studied extensively in student nurses [7, 8]. Nursing students tend to report higher levels of stress outcome than other student populations in many countries [9, 10]. In China, up to 28.6% of nursing students during their education suffer from some type of mental health problem, such as distress and anxiety [11].

The research has showed that perceived stress of Chinese nursing students was relatively high. However, until recently, there was no recognized scale used to evaluate the stress of nursing students in Mainland China. The Chinese version of the Perceived Stress Questionnaire (CPSQ) has been confirmed as valid and reliable instruments in nursing students [12], but the CPSQ was not widely used in China. The quality of measurement tools used internationally still needed to be further developed. Currently, many scales are used to assess the stress of the student nurses across a range of international settings, such as the Index of Sources of Stress in Nursing Students (ISSN) [13], the Student Nurse Stress Index Scale (SNSI) [14], the Stressors in Student Nursing Scale (SINS) [15], the Perceived Stress Scale (PSS) [16], and the Perceived Stress Questionnaire (PSQ) [17]. The Chinese version of PSS (CPSS) is commonly used as a measurement stress outcome, i.e., to assessing individuals' stress levels in China, and it has been proved to have good reliability and validity in policewomen [18] and nursing students [19]. The CPSS was designed primarily for use in the general adult population and was not specifically designed to measure the stress levels reported by nursing students [20]. The CPSS remains, however, the most widely used, robust, and acceptable measure of stress outcome available in China. Foreign studies have proved that the Student Nurse Stress Index Scale (SNSI) and the Stressors in Student Nursing Scale (SINS) all have good reliability and validity. The Chinese version of SINS (SINS-CHI) and the Chinese version of SNSI (SNSI-CN) also have been, respectively, studied for their reliability and validity, but the effect and consistency of the two scales have not been compared. In this study, the characteristics, application effects, and consistency of the two methods were studied and evaluated to provide reference for the research of the stress assessment tools for nursing students.

## 2. Background

With the reform and transformation of nursing as a clinical-academic discipline, nursing students are facing great pressure and challenges [21]. Nursing is a specialty that requires students to integrate highly detailed clinical knowledge with skilled, considered practical application ability [22]. Our review of a diffuse literature suggests that student nurses experience three main sources of stress or demand, i.e., academic, clinical practice, and personal concerns [23, 24].

Academic concerns associated with emotional distress in student nurses include fear of exam failure, competitive environment, and heavy workload [25]. Poor performance and failing to achieve expected results or meeting family expectations in this regard can make the students experience emotional distress and be at risk for developing symptoms of anxiety [26]. Regarding the clinical environment, student nurses tend to report stress outcomes associated with the demand of attempting to apply theory within practice [27]. Issues such as feeling unprepared for practice, fear of making a mistake, issues related to death and dying, witnessing pain and suffering, problematic interpersonal relationships with clinical teachers and nursing staff, being observed and evaluated, communicating with physicians, and lack of familiarity with the hospital environment are commonly reported stressful demands. Emotional distress in student nurses is often associated with personal concerns regarding financial issues and limited leisure time [28].

In recent years, in order to meet the growing demand of medical institutions at all levels for higher nursing talents, medical colleges in China have begun to increase the number of undergraduate nursing students enrolled year by year, not only paying more attention to the cultivation of professional knowledge and operational skills of nursing students, but also paying attention to the development of undergraduate nursing students' physical and mental health [29]. The evidence suggests that student nurses experience high levels of emotional distress, particularly during the early and later stages of their education. Stress is considered as normal and adaptive in a range of circumstances [30], and mild stress can make the body in a tense state and improve learning efficiency [31]. However, excessive levels of stress, i.e., distress, may lead to physical and mental health problems, such as anxiety, depression, and sleep problems, and may affect students' academic performance [32].

Nursing students' coping with stress is a dynamic process [33]. The most important thing is to find out the source of stress so as to formulate coping strategies to help them relieve and cope with stress [34]. Therefore, the timely development of stress assessment tools is conducive to further understanding of the current situation of pressure and exploring the source of pressure. The Chinese versions of SNSI (SNSI-CHI) and SINS (SINS-CN) have been confirmed as valid and reliable instruments, and the SNSI and SINS are specifically used to measure the source and the level of stress for nursing students [35, 36]. To enrich and develop instruments for assessing stress of the nursing students in China, this study is aimed at further testing and comparing the characteristics and the application effect of the two stress instruments to verify suitable instruments for using in Chinese nursing students through an in-depth comparative analysis.

## 3. Aim and Objective

This study is aimed at testing and comparing the reliability and validity, including sensitivity and specificity, of the two nursing students' stress instruments, the SNSI-CHI and the SINS-CN in Chinese nursing student, and describing the stress status of nursing students in China.

## 4. Methods

### 4.1. Research Design

A cross-sectional survey was conducted in two Nursing Schools in Henan Province of central China from August 2017 to January 2018. These institutions provide undergraduate nursing education which is regulated by the educational objectives of the Henan Province in China.

### 4.2. Participants

One thousand and one hundred students were initially given with questionnaires, of which 1076 were completed and returned. Potential study participants met the following criteria before being enrolled into this study: (1) participants were ≥16 years old, (2) had experienced clinical practice for ≥1 month, and (3) provided consent to participate. Exclusion criteria included students who were absent during the survey period.

### 4.3. Ethical Considerations

This study was approved by the Ethics Committee of Zhengzhou University (Ethical No: 2017-04-N008). These nursing students were given the significance and the purpose of study and obtained the verbal informed consent of the participants at the beginning of the interview. This had been approved by the ethics committee, as well as the approval for participants under the age of 18 to participate and consent on their own behalf. Participation was voluntary, and confidentiality and anonymity were assured throughout the study.

### 4.4. Instruments

#### 4.4.1. General Questionnaire

The general questionnaire was designed by researchers on the basis of literature to include demographic variables such as age, gender, grade, hometown, household income, height, weight, body mass index (BMI), student leader or not, and period of clinical practice.

#### 4.4.2. The Student Nurse Stress Index Scale (SNSI-CHI)

The Student Nurse Stress Index Scale (SNSI) [14] was developed to provide an improved, robust measure of nursing student's sources of stress. It consists of 22 items clustered into four factors: academic load (items 1, 2, 3, 8, 14, 18, and 20), clinical concerns (items 13, 14, 16, 17, 18, and 19), interface worries (items 4, 5, 6, 7, 15, 21, and 22), and personal problems (items 9, 10, 11, and 12). SNSI uses a five-point Likert scale ranging from 1 (not stressful) to 5 (extremely stressful). The total score ranges from 22 to 110, and the higher scores demonstrate a higher level of demand or sources of stress. The original SNSI had been translated into Chinese version (SNSI-CHI) and proved that it had good reliability and validity in China [35].

#### 4.4.3. The Stressors in Student Nursing Scale (SINS-CN)

The Stressors in Student Nursing Scale (SINS) is a 43 item, self-administered questionnaire, which was developed with nursing students in Scotland in a longitudinal study [15]. It investigated, with a five-point Likert type response, how stressful are various aspects of being a nursing student. The scales for each item run from 1 = “not stressful” to 5 = “extremely stressful.” The original SINS had been back translated into the Chinese version (SINS-CN) with simplified Chinese characters and deleted some items during its application in China (items 5, 6, 11, 16, 19, 21, 24, 29, 39, 40, and 42). The result of the study showed that it had good reliability and validity [36]. The scale identifies four factors or sources of stress in the student nurse, namely, clinical (items 7, 8, 9, 10, 12, 13, 14, 15, 17, 20, 32, and 43), confidence (items 2, 23, 27, 30, 31, 34, 36, and 38), education (items 1, 3, 4, 18, and 33), and finance (items 22, 25, 26, 28, 35, 37, and 41). The total score ranges from 32 to 160, and the higher scores demonstrate a higher level of demand or sources of stress.

#### 4.4.4. Perceived Stress Scale (PSS)

The Perceived Stress Scale (PSS), which is the most widely used psychological scale as a stress outcome, was developed by Cohen in 1983, and it has shown sufficient reliability and validity [16]. The items of PSS are easily understandable and scorable, which when translated to traditional Chinese and tested in China, and showed good reliability and validity with a Cronbach's *α* of 0.78 [19]. The Chinese version consists of 14 items and two dimensions, seven investigate respondent sense of being out of control (items 4, 5, 6, 7, 9, 10, and 13) while others relate to tension (items 1, 2, 3, 8, 11, 12, and 14). Participants were required to answer each question using a five-point Likert scale score ranging from 0 (never) to 4 (very often). Total scores ranged from 0 to 56, and participants with higher scores had higher perceived cognitive and emotional stress levels.

### 4.5. Data Collection

A stratified cluster random sampling of nursing student was recruited from Schools of Nursing in medical colleges or universities in Henan Province from August 2017 to January 2018. Two medical colleges or universities were randomly selected from five in Henan Province and were then stratified them into four layers according to grade (freshman, sophomore, junior, and senior); then, six classes were randomly selected in each layer.

Survey stations were established in the classrooms in School of Nursing, and the nursing students were invited to approach. A one-to-one, face-to-face data collection method was then used in this study. The paper questionnaire used was completed or answered voluntarily by the participants in the classroom settings. Data collection was both private and anonymous.

During this survey, investigators who administered the questionnaire were provided with uniform standardized training before conducting this investigation. All the participants were informed about the aim and the procedures of the study. Participants finished the questionnaires on their own, and assistance was provided if participants had difficulty to read the questions and write their answers independently. Afterward, the questionnaires completed less than 80% or with low writing quality were excluded from this survey. In addition, 30 nursing students who agreed to take the survey again were selected for the retest two weeks later.

### 4.6. Data Analysis

All analyses were carried out using SPSS 21.0 statistical program (International Business Machines Corporation, Armonk, New York, USA). Data of each student participant who have completed the three self-administered questionnaires were analyzed using descriptive statistics, Pearson correlation coefficient, and multiple regression analyses. Significance was set at a level of 0.05.

The reliability of the SNSI-CHI and SINS-CN was established using the consistency and stability [37]. The internal consistency of the SNSI-CHI and SINS-CN were estimated using Cronbach's *α* and the Guttman Split-Coefficient. Item-to-total (estimated by Pearson's rank correlation coefficients) was used to test the homogeneity of the scales. Stability of SNSI-CHI and SINS-CN was estimated by test-retest correlation coefficient (intraclass correlation coefficient, ICC).

The content validity of the SNSI-CHI and SINS-CN was calculated using item level (I-CVI) and scale level (S-CVI). I-CVI was estimated by dividing the sum of experts with a score of 3 or 4 by the number of experts, and S-CVI/Ave (average) was the average of the I-CVIs for all items on the scale [38]. Each expert gave a mark to each item with four grades: 1 = not related; 2 = weak correlation; 3 = strong correlation; and 4 = very relevant. The “not related” and “weak correlation” options yielded a score of 0, and the “strong correlation” and “very relevant” options yielded a score of 1. The CVI determined by five experts that was greater than 0.80 was judged to show good content validity [39].

The construct validity of the SNSI-CHI and SINS-CN was established using exploratory factor analyses (EFA); concurrent validity was estimated by Pearson's rank correlation coefficients between total score of SNSI-CHI and CPSS and their factors and between the total score of SINS-CN and CPSS and their factors.

The predictive validity of the SNSI-CHI and SINS-CN was estimated using receiver operator characteristic (ROC) curves, measures of sensitivity and specificity, and the Youden's index. Receiver operator characteristic (ROC) curves, sensitivity, specificity, positive predictive value (PPV) and negative predictive value (NPV), and the Youden's index were estimated to find suitable cut-off points of SNSI-CHI and SINS-CN [40]. The CPSS was regarded as the criterion. The total score ≥ 27 of CPSS indicates a high level of stress; and the total score < 27 of CPSS indicates a middle-low level of stress [41].

## 5. Results

### 5.1. Participant Characteristics

Initially, 1100 nursing students were recruited to the study, and a total of 1076 students completed the questionnaires giving an overall response rate of 97.80%. The demographic characteristics are displayed in Table 1. In terms of the SNSI-CHI and SINS-CN scores, the result (Table 2) showed that the scores of female undergraduate nursing students were significantly lower than that of male nursing students, the scores of senior nursing students were higher than that of other grades, and the scores of undergraduate nursing students as student leaders were lower than that of ordinary students.

### 5.2. Reliability

The values of homogeneity of the SNSI-CHI, which serve as a measure of reliability were yielded with Cronbach's *α* coefficient of 0.90 and Guttman Split-Coefficient of 0.83, and the four factors of SNSI-CHI yielded a Cronbach's *α* of 0.75, 0.74, 0.72, and 0.71, in turn. Homogeneity of the SINS-CN was indicated with a Cronbach's *α* coefficient of 0.93 and Guttman Split-Coefficient of 0.89, and the four factors of SINS-CN yielded a Cronbach's *α* of 0.76, 0.69, 0.70, and 0.70, respectively.

There was no single item that if deleted would improve the overall Cronbach's *α* for SNSI-CHI (Table 3) or SINS-CN (Table 4). The item-to-total correlations of SNSI-CHI ranged between *r* = 0.35 and *r* = 0.66, where the average correlation was *r* = 0.59 (Table 3), and the item-to-total correlations of SINS-CN ranged between *r* = 0.19 and *r* = 0.61, where the average correlation was *r* = 0.50 (Table 4).

The SNSI-CHI and SINS-CN were administered to all students, and 30 students readministered the SNSI-CHI and SINS-CN after two weeks to determine the test-retest reliability. The ICC of the SNSI-CHI was adequate at 0.99 (95% CI, 0.99~1.00; *P* < 0.001), and the ICC of the SINS-CN was also adequate at 0.95 (95% CI, 0.92~0.96; *P* < 0.001).

### 5.3. Validity

The SNSI-CHI and SINS-CN were assessed by the five experts, I-CVI of the SNSI-CHI and SINS-CN ranged from 0.80 to 1.00, the S-CVI/Ave of the SNSI-CHI yielded a value of 0.95, and the S-CVI/Ave of the SINS-CN yielded a value of 0.83, which served as a measure of content validity.

Exploratory factor analysis of the SNSI-CHI indicated that the Kaiser-Meyer-Olkin (KMO) was 0.963, and the Bartlett's Test of Sphericity was 10389.365, with significant statistical significance (*P* < 0.01). Four factors were extracted with eigenvalues > 1.00, after the principal component analysis and varimax orthogonal rotation. The four extracted factors explained 75.013% of the total variance. The factor loadings and the values of communality appear in Table 3. The exploratory factor analysis results of the SINS-CN indicated that the Kaiser-Meyer-Olkin (KMO) was 0.942, and the Bartlett's Test of Sphericity was 14763.670, with significant statistical significance (*P* < 0.01). Four factors were extracted with eigenvalues > 1.00, after the principal component analysis and varimax orthogonal rotation. The four extracted factors explained 64.835% of the total variance. The factor loadings and communalities are displayed in Table 4.

The Pearson's rank correlation coefficients between the total score of SNSI-CHI and CPSS and their factors showed that the two instruments were significantly correlated (*P* < 0.01, *r* = 0.10 to 0.42). The SINS-CN and the CPSS and their factors were significantly correlated (*P* < 0.01, *r* = 0.08 to 0.45) (see Table 5).

### 5.4. Sensitivity and Specificity

**Figure 1:**
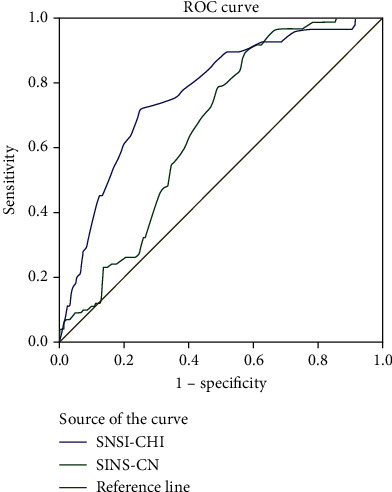
The receiver operator characteristic (ROC) curve of the SASE-CHI and SINS-CN with CPSS as a criterion (*n* = 1076). SNSI-CHI: the Chinese version of the Student Nurse Stress Index; SINS-CN: the Chinese version of the Stressors in Student Nursing Scale; CPSS: the Chinese Perceived Stress Scale.

The result of the ROC curves showed that the area under the ROC curve of SNSI-CHI for the optimal cut-point was 0.77 (95% CI: 0.73~0.82, *P* < 0.001), and the area under the ROC curve of SINS-CN for the optimal cut-point was 0.66 (95% CI, 0.62~0.70, *P* < 0.001). The sensitivity was 71.7%, and specificity was 75.1% for the student nurse based on a cutoff value of 65 of the SNSI-CHI. The sensitivity was 78.8%, and specificity was 51.2% for the student nurse based on a cutoff value of 98 of the SINS-CN. The specificity and sensitivity were calculated based on the ROC curve generated by the data obtained from our study (Figure 1).

## 6. Discussion

The finding of this cross-sectional survey was that both the SNSI-CHI and SINS-CN were homogenous and stable instruments for the evaluation of stress in nursing students. At present, the SNSI has been used in the United Kingdom [14], the United States [42], India [43], Turkey [44], and other countries, and the SINS has been used in Scotland [15] and Hong Kong [45]. It had been proved that the two scales are reliable and valid instruments for use with nursing students.

In this study, the stress level of nursing students in China is on the high side. The mean score of all items of SNSI-CHI was 58.37 and SINS-CN was 85.37; the stress level of nursing students in China was slightly higher than that in foreign countries. The reason may be influenced by traditional Chinese culture and the form of nurses' education, and nursing students in China have a low social status and face competition for employment [46, 47]. Undergraduate female nursing students with higher scores than male nursing students, which may be due to the fact that the nursing industry is dominated by women, coupled with a high workload environment [48]. The scores of senior nursing students was higher than that of other grades, which may be due to the fact that senior nursing students are about to enter the clinical practice stage and will face various uncertainties in the future [29]. As for whether to be a student leader or not, the scores of as student leaders was lower than that of ordinary students. This may be because nursing students who are student leaders have more autonomous decision-making power and more opportunities to communicate with teachers and develop the ability to solve problems. On the other hand, there are more opportunities for student cadres to contact with their classmates, and they have good interpersonal relationship [49]. Since the prevalence of emotional distress levels of stressful demand in nursing students is high, it is important to develop reliable, valid, and acceptable screening instruments to identify the nursing students who are at risk for high source of stress level.

The Cronbach's *α* coefficient and test-rests were used to evaluate the reliability of this study. The coefficients of Cronbach's *α* and Guttman Split-Coefficients of the SNSI-CHI and SINS-CN were found to be satisfactory (*>*0.70) [50]. Studies which have investigated the stress levels of student nurses and the influencing factors have reported the Cronbach's *α* coefficient of SNSI as 0.79 in India [43] and 0.89 in California [42] with 154 student nurse. The Cronbach's *α* coefficient of SINS was reported as 0.82 in a sample of nursing students in Scotland [15] and 0.83 in a sample of nursing students in Hong Kong [45].

All item-to-total correlations, in this study, were statistically significant and in agreement with the recommended standard. Thus, the results indicate that the two scales demonstrate good homogeneity. The ICC of the SNSI-CHI was adequate at 0.99, and the ICC of the SINS-CN was also adequate at 0.95. With the acceptable range of the ICC established as 0.81~1.00, both measures almost perfectly fit this criterion [51, 52]. The obtained coefficients of the test-retest ICCs of SNSI-CHI and SINS-CN showed both instruments have very good stability. This all suggests that SNSI-CHI and SINS-CN are credible scales with a higher level of homogeneity and stability compared to other studies.

The S-CVI of SNSI-CHI and SINS-CN exceeded 0.80, indicating a good content validity compared against recommendations of a minimum S-CVI score of 0.80 [53]. Our experts were harmonious because the calculated values were higher than the minimum value, and the S-CVI/Ave of SNSI-CHI was slightly higher than the S-CVI/Ave of SINS-CN. The Pearson's rank correlation, which serves as measures of concurrent validity, ranged from 0.10 to 0.42 for SNSI-CHI and CPSS, and 0.08 to 0.45 for SINS-CN and CPSS. In China, CPSS is also used as a criterion to test the reliability and validity of SNSI-CHI [35]. The correlation of CPSS with SNSI-CHI was 0.33~0.58, which was slightly higher than in this study. All correlations were significantly correlated, but the correlation coefficient between SNSI-CHI and CPSS was weaker compared to the correlation coefficient between SINS-CN and CPSS.

The construct validity of the SNSI-CHI and SINS-CN were estimated with an exploratory factor analysis. Each item should beyond load onto its hypothesized factor beyond a cut-off (>0.40), while having low load values on other common factors. The cumulative variance contribution ratio of the common factors should be at least 40%. The EFA revealed four factors with an eigenvalue greater than 1.00 of SNSI-CHI, namely, academic load, 5 items; clinical concerns, 6 items; interface worries, 7 items; and personal problems, 4 items. This is consistent with the previous studies [14, 44]. The items for factor 1 and factor 2 were almost the same as in the testing studies of the original version, except that item 14 (too much responsibility), item 18 (Atmosphere created by teaching staff), and item 20 (I am not sure what is expected of me) which belongs to both factor 1 and factor 2. In our study, item 20 only belongs to factor 1, while item 14 and item 18 only belong to factor 2. The differences in the factors might be due to the participants' different cultural background, living habits, and discipline education. The EFA of SINS-CN also revealed four factors with an eigenvalue greater than 1.00: clinical, 12 items; finance, 7 items; confidence, 8 items; and education, items. The findings are consistent with Watson et al. [15, 36, 45]. The EFA of SNSI-CHI and SINS-CN in the present study yielded a similar factor structure with the previous studies.

High sensitivity and specificity values indicate that the chance of determination of stress by the SNSI-CHI and the SINS-CN test is better. In our study, the findings showed that the sensitivity and specificity of both the SNSI-CHI and the SINS-CN are high for Chinese nursing students. The area under the ROC curve for the optimal cut-point was 0.77 of SNSI-CHI and 0.66 of SINS-CN. The area under the curve can best represent the effect of the scale's detection results with an expected range between 0.5 and 1.0, with higher value indicating the better of the effect [54]. The results of the ROC curves showed that the area under the ROC curve of SNSI-CHI was larger than SINS-CN in this study. This showed that the sensitivity and specificity of SNSI-CHI was higher than SINS-CN.

The present study was the first study which explored the cut-off point of SNSI-CHI and SINS-CN. The cut-off point of the Chinese version of SNSI-CHI was 65, and 98 for the Chinese version of SINS-CN. However, additional follow-up studies are required to further confirm this. The nursing students whose score exceed the cut-off point were identified as experiencing a high level of source of stress, approximately one in ten (114/1076 = 10.59%) of respondents experienced high levels of stressful demand in our study. Therefore, it is important to explore the cutoff scores to help identify those who are at greater risk of high level of source of stress, and follow-up studies are needed.

Both SNSI-CHI and SINS-CN are self-report scales which are used to measure the sources of stress facing nursing students and with demonstrated strong pertinence. At the same time, it pays more attention to the negative impact of stress on nursing students. A self-report instrument can assess the potential of the source of stress. However, because of strong subjectivity, the self-reported instrument may underestimate or overestimate the source of stress of the individual. So, when using self-reported scales, health professionals should observe behaviors of the individuals. The characteristic of SINS-CN is that it can measure the change of stress level in different period, and the source of pressure can be measured at the same time, which has high consistency across time. In addition, the SINS-CN content comprehensively investigates the stress status of nursing students, by evaluating in clinical, confidence, economic, and education dimensions. However, there are many items of SINS-CN, which it will take up more time in the survey process. Compared with SNSI-CHI, the SINS-CN entries are more, so the workload is relatively large. Both of the two scales all have their own characteristics, most important is that the user can choose the best instrument according to the specific situation.

## 7. Study Limitations

This study has the following limitations. First, the sample was limited to Henan Province, and only 30 students were conducted to test-retest reliability; this may have an influence on the generalizability of the result. A more diverse sample should be included in follow-up studies. Second, our study only assessed the negative influence of stress on nursing students, but did not evaluate the positive effects of stress. Therefore, the future research can be developed to capture such positive antecedents and outcomes. Despite these limitations, the results of the reliability and validity tests of the SNSI-CHI and SINS-CN show similarities with earlier testing studies.

## 8. Conclusions

As a result of the findings obtained from the validity and reliability studies of the SNSI-CHI and SINS-CN, we report that the two scales are valid and reliable for evaluating the stress of student nurses in China. The SNSI-CHI demonstrated marginal advantage over the SINS-CN. The SNSI-CHI is short, easily understood, and with clear applicability for the nursing students, so the SNSI-CHI is more acceptable for the users in China. However, further studies to test the reliability, validity, sensitivity, and specificity in different geographical populations in China should be encouraged.

## Figures and Tables

**Table 1:** Characteristics of the sample (*n* = 1076).

Variables	Classify	Number	Percentage
Age in years	17-21	648	60.2
	22-25	428	39.8

Gender	Male	80	7.4
	Female	996	92.6

Grade	Year one	248	23.0
	Year two	245	22.8
	Year three	293	27.2
	Year four	290	27.0

Hometown	Rural areas	795	73.9
	Urban areas	281	26.1

Household income status	≥30,000 RMB/month	27	2.5
	≥10,000 RMB/month	191	17.8
	>5000 RMB/month	759	70.5
	≤5000 RMB/month	99	9.2

Had been a class leaders	Yes	278	25.8
	No	798	74.2

Having an interest in nursing profession	Yes	343	31.9
	No	733	68.1

Duration of clinical practice	1-3	467	43.4
	4-6	609	56.6

**Table 2:** Means and standard deviations of the scales of SNSI-CHI, SINS-CN, and CPSS.

Variables	Range of scores	Mean	Standard deviation
SNSI-CHI	22-102	58.37	13.66
SINS-CN	32-135	85.37	18.73
CPSS	0-46	25.83	5.95

**Table 3:** Item-to-total score Pearson's rank correlation coefficients for SNSI-CHI and factor loading of SNSI-CHI items from factor analysis (*n* = 1076).

Items	Item-to-total correlation coefficient (*r*)	Cronbach's *α* if item deleted	F1	F2	F3	F4	Communality
S1	0.621^※^	0.879	**0.733**	-0.469	0.103	0.009	0.799
S2	0.664^※^	0.878	**0.711**	0.323	0190	0.117	0.740
S3	0.656^※^	0.878	**0.748**	-0.468	0.190	0.117	0.779
S4	0.615^※^	0.879	0.360	0.026	**0.848**	0.219	0.803
S5	0.599^※^	0.879	0.178	0.047	**0.843**	0.042	0.810
S6	0.618^※^	0.879	0.100	0.104	**0.825**	0.374	0.783
S7	0.351^※^	0.898	0.248	0.016	**0.801**	0.033	0.719
S8	0.660^※^	0.878	**0.715**	-0.262	0.097	0.062	0.788
S9	0.551^※^	0.881	0.310	0.291	0.070	**0.650**	0.474
S10	0.550^※^	0.881	0.121	0.125	0.189	**0.725**	0.554
S11	0.421^※^	0.884	0.311	0.377	0.090	**0.700**	0.515
S12	0.528^※^	0.881	0.388	0.381	0.095	**0.727**	0.537
S13	0.601^※^	0.880	0.352	**0.827**	0.165	0.268	0.812
S14	0.634^※^	0.878	0.041	**0.830**	0.142	0.081	0.873
S15	0.655^※^	0.878	0.115	0.185	**0.841**	0.151	0.812
S16	0.611^※^	0.879	0.224	**0.828**	0.304	0.098	0.837
S17	0.496^※^	0.883	0.153	**0.820**	0.378	0.109	0.816
S18	0.453^※^	0.885	0.080	**0.835**	0.198	0.222	0.844
S19	0.584^※^	0.880	0.212	**0.821**	0.082	0.306	0.832
S20	0.426^※^	0.888	**0.733**	0.154	0.103	0.046	0.806
S21	0.633^※^	0.878	0.082	0.095	**0.814**	0.360	0.761
S22	0.593^※^	0.886	0.149	0.145	**0.846**	0.366	0.809

^※^ *P* < 0.01; SNSI-CHI: the Chinese Version of the Student Nurse Stress Index Scale; S1-S22: item 1-item 22; S1-S22: item 1-item 22; factor 1 (academic load, 6 items), factor 2 (clinical concerns, 5 items), factor 3 (interface worries), and factor 4 (personal problems, 4 items).

**Table 4:** Item-to-total score Pearson's rank correlation coefficients for SINS-CN and factor loading of SINS-CN items from factor analysis (*n* = 1076).

Items	Item-to-total correlation coefficient (*r*)	Cronbach's *α* if item deleted	F1	F2	F3	F4	Communality
S1	0.547^※^	0.921	0.216	0.148	0.114	**0.800**	0.854
S2	0.198^※^	0.924	0.048	0.202	**0.532**	0.051	0.639
S3	0.424^※^	0.922	0.135	0.298	0.007	**0.735**	0.776
S4	0.558^※^	0.921	0.248	0.065	0.269	**0.874**	0.864
S7	0.545^※^	0.921	**0.869**	0.122	0.168	0.291	0.818
S8	0.343^※^	0.926	**0.745**	0.007	0.193	0.228	0.809
S9	0.599^※^	0.921	**0.728**	0.260	0.378	0.101	0.830
S10	0.420^※^	0.923	**0.753**	0.230	0.372	0.234	0.669
S12	0.550^※^	0.921	**0.782**	0.366	0.001	0.281	0.788
S13	0.615^※^	0.921	**0.754**	0.311	0.309	0.271	0.691
S14	0.490^※^	0.922	**0.805**	0.234	0.345	0.092	0.746
S15	0.393^※^	0.924	**0.574**	0.269	0.237	0.161	0.802
S17	0.486^※^	0.922	**0.902**	0.312	0.206	0.064	0.58
S18	0.470^※^	0.922	0.380	0.011	0.156	**0.790**	0.755
S20	0.596^※^	0.921	**0.736**	0.300	0.155	0.212	0.835
S22	0.593^※^	0.921	0.160	**0.888**	0.101	0.305	0.869
S23	0.609^※^	0.921	0.109	0.330	**0.757**	0.188	0.801
S25	0.574^※^	0.921	0.287	**0.891**	0.145	0.190	0.884
S26	0.565^※^	0.921	0.226	**0.745**	0.219	0.150	0.715
S27	0.478^※^	0.922	0.161	0.230	**0.684**	0.004	0.838
S28	0.480^※^	0.922	0.023	**0858**	0.016	0.279	0.812
S30	0.473^※^	0.922	0.245	0.140	**0.691**	0.049	0.718
S31	0.608^※^	0.921	0.302	0.201	**0.664**	0.121	0.768
S32	0.643^※^	0.920	**0.648**	0.306	0.261	0.218	0.714
S33	0.560^※^	0.921	0.110	0.276	0.275	**0.785**	0.797
S34	0.432^※^	0.922	0.257	0.129	**0.616**	0.359	0.684
S35	0.469^※^	0.922	0.383	**0.878**	0.277	0.288	0.823
S36	0.510^※^	0.921	0.219	0.369	**0.771**	0.029	0.798
S37	0.473^※^	0.922	0.262	**0.900**	0.033	0.148	0.852
S38	0.460^※^	0.922	0.120	0.271	**0.612**	0.01	0.767
S41	0.603^※^	0.921	0.373	**0.888**	0.056	0.177	0.895
S43	0.564^※^	0.921	**0.705**	0.177	0.026	0.305	0.763

^※^ *P* < 0.01; SINS-CN: the Chinese Version of the Stressors in Student Nursing Scale; S1-S43: item 1-item 43; S1-S43: item 1-item 43; factor 1 (clinical, 12 items), factor 2 (finance, 7 items), factor 3 (confidence, 8 items), and factor 4 (education, 5 items).

**Table 5:** The Pearson's rank correlation coefficients of three scales (*n* = 1076).

	SNSI-C	SN1	SN2	SN3	SN4	SINS-C	SI1	SI2	SI3	SI4	CPSS	C1	C2
SNSI-C	1												
SN1	0.819^※^	1											
SN2	0.828^※^	0.556^※^	1										
SN3	0.874^※^	0.628^※^	0.604^※^	1									
SN4	0.704^※^	0.456^※^	0.504^※^	0.493^※^	1								
SINS-C	0.838^※^	0.696^※^	0.741^※^	0.712^※^	0.542^※^	1							
SI1	0.730^※^	0.599^※^	0.668^※^	0.616^※^	0.458^※^	0.897^※^	1						
SI2	0.650^※^	0.505^※^	0.550^※^	0.620^※^	0.384^※^	0.784^※^	0.599^※^	1					
SI3	0.717^※^	0.597^※^	0.645^※^	0.587^※^	0.544^※^	0.818^※^	0.645^※^	0.580^※^	1				
SI4	0.614^※^	0.654^※^	0.492^※^	0.501^※^	0.309^※^	0.746^※^	0.628^※^	0.503^※^	0.505^※^	1			
CPSS	0.387^※^	0.335^※^	0.317^※^	0.295^※^	0.330^※^	0.394^※^	0.333^※^	0.241^※^	0.384^※^	0.291^※^	1		
C1	0.174^※^	0.158^※^	0.153^※^	0.096^※^	0.186^※^	0.152^※^	0.104^※^	0.047^※^	0.237^※^	0.078^※^	0.752^※^	1	
C2	0.422^※^	0.359^※^	0.337^※^	0.352^※^	0.330^※^	0.451^※^	0.400^※^	0.313^※^	0.366^※^	0.360^※^	0.844^※^	0.280^※^	1

SNSI-C: the Chinese version of the Student Nurse Stress Index. SN1, SN2, SN3, and SN4 are factor 1, factor 2, factor 3, and factor 4 of SNSI, respectively. SINS-C: the Chinese version of the Stressors in Student Nursing Scale. SI1, SI2, SI3, and SI4 are factor 1, factor 2, factor 3, and factor 4 of SINS-C, respectively. CPSS: the Chinese Perceived Stress Scale. C1 and C2 are factor 1 and factor 2 of CPSS, respectively. ^※^*P* < 0.01.

## Data Availability

The data used to support the findings of this study are available from the corresponding author upon request.

## References

[1] Smith M. J., Selye H. (1979). Stress: reducing the negative effects of stress. *American Journal of Nursing*.

[2] Folkman S., Lazarus R. S. (1985). If it changes it must be a process: study of emotion and coping during three stages of a college examination. *Journal of Personality and Social Psychology*.

[3] Lazarus R. S. (1993). Coping theory and research: past, present, and future. *Psychosomatic Medicine*.

[4] Seib C., Porter-Steele J., Ng S.-K. (2018). Life stress and symptoms of anxiety and depression in women after cancer: the mediating effect of stress appraisal and coping. *Psycho-Oncology*.

[5] Grobecker P. A. (2016). A sense of belonging and perceived stress among baccalaureate nursing students in clinical placements. *Nurse Education Today*.

[6] Fornés-Vives J., Garcia-Banda G., Frias-Navarro D., Rosales-Viladrich G. (2016). Coping, stress, and personality in Spanish nursing students: a longitudinal study. *Nurse Education Today*.

[7] Jones M. C., Johnston D. W. (1997). Distress, stress and coping in first-year student nurses. *Journal of Advanced Nursing*.

[8] Jones M. C., Johnston D. W. (2000). Reducing distress in first level and student nurses: a review of the applied stress management literature. *Journal of Advanced Nursing*.

[9] Aysola J., Barg F. K., Martinez A. B. (2018). Perceptions of factors associated with inclusive work and learning environments in health care organizations: a qualitative narrative analysis. *JAMA Network Open*.

[10] McCarthy B., Trace A., O’Donovan M. (2018). Nursing and midwifery students' stress and coping during their undergraduate education programmes: an integrative review. *Nurse Education Today*.

[11] Liu M., Jia Q. (2003). Comparison of mental health status and social support system of nursing students from different place. *Nursing Journal of Chinese People's Liberation Army*.

[12] Luo Y., Gong B., Meng R. (2018). Validation and application of the Chinese version of the Perceived Stress Questionnaire (C-PSQ) in nursing students. *PeerJ*.

[13] Gibbons C., Dempster M., Moutray M. (2009). Index of sources of stress in nursing students: a confirmatory factor analysis. *Journal of Advanced Nursings*.

[14] Jones M. C., Johnston D. W. (1999). The derivation of a brief Student Nurse Stress Index. *Work & Stress*.

[15] Deary I. J., Watson R., Hogston R. (2003). A longitudinal cohort study of burnout and attrition in nursing students. *Journal of Advanced Nursings*.

[16] Cohen S., Kamarck T., Mermelstein R. (1983). A global measure of perceived stress. *Journal of Health and Social Behavior*.

[17] Levenstein S., Prantera C., Varvo V. (1993). Development of the Perceived Stress Questionnaire: a new tool for psychosomatic research. *Journal of Psychosomatic Research*.

[18] Wang Z., Chen J., Boyd J. E. (2011). Psychometric properties of the Chinese version of the Perceived Stress Scale in policewomen. *PLoS One*.

[19] Yang T., Huang H. (2003). An epidemiological study on stress among urban residents in social transition period. *Chinese Journal of Epidemiology*.

[20] Liu Y., Li T., Guo L., Zhang R., Feng X., Liu K. (2017). The mediating role of sleep quality on the relationship between perceived stress and depression among the elderly in urban communities: a cross-sectional study. *Public Health*.

[21] Goff A. M. (2011). Stressors, academic performance, and learned resourcefulness in baccalaureate nursing students. *International Journal of Nursing Education Scholarship*.

[22] Gazzaz Z. J., Baig M., al Alhendi B. S. M. (2018). Perceived stress, reasons for and sources of stress among medical students at Rabigh Medical College, King Abdulaziz University, Jeddah, Saudi Arabia. *BMC Medical Education*.

[23] Pryjmachuk S., Richards D. A. (2007). Predicting stress in pre-registration nursing students. *British Journal of Health Psychology*.

[24] Jimenez C., Navia-Osorio P. M., Diaz C. V. (2010). Stress and health in novice and experienced nursing students. *Journal of Advanced Nursing*.

[25] Ratanasiripong P., Park J. F., Ratanasiripong N., Kathalae D. (2015). Stress and anxiety management in nursing students: biofeedback and mindfulness meditation. *Journal of Nursing Education*.

[26] Donough G., Van der Heever M. (2018). Undergraduate nursing students' experience of clinical supervision. *Curationis*.

[27] Chan Z. C. Y., Cheng W. Y., Fong M. K. (2019). Curriculum design and attrition among undergraduate nursing students: a systematic review. *Nurse Education Today*.

[28] Dendle C., Baulch J., Pellicano R. (2018). Medical student psychological distress and academic performance. *Medical Teacher*.

[29] Wu M., Sun X. (2019). The relationship between undergraduate nurse pressure and mental health: the mediating function of psychological resilience. *Chinese Journal of Mental Health*.

[30] Collet J., de Vugt M. E., Schols J. M. G. A., Engelen G. J. J. A., Winkens B., Verhey F. R. J. (2018). Well-being of nursing staff on specialized units for older patients with combined care needs. *Journal of Psychiatric and Mental Health Nursing*.

[31] Wang A. W.-T., Bouchard L. C., Gudenkauf L. M. (2018). Differential psychological effects of cognitive-behavioral stress management among breast cancer patients with high and low initial cancer-specific distress. *Journal of Psychosomatic Research*.

[32] Alsaggaf M., Wali S., Merdad R., Merdad L. (2016). Sleep quantity, quality, and insomnia symptoms of medical students during clinical years. Relationship with stress and academic performance. *Saudi Medical Journal*.

[33] Labrague L. J., McEnroe–Petitte D. M., de Los Santos J. A. A., Edet O. B. (2018). Examining stress perceptions and coping strategies among Saudi nursing students: a systematic review. *Nurse Education Today*.

[34] Rafati F., Nouhi E., Sabzevari S., Dehghan-Nayeri N. (2017). Coping strategies of nursing students for dealing with stress in clinical setting: a qualitative study. *Electronic Physician*.

[35] Guo L., Suyuan Y. U., Zhu Y. (2018). Reliability and validity of the Chinese version of the student nurse stress index scale (SNSI-CHI). *Chinese Journal of Behavioral Medicine and Brain Science*.

[36] Watson R., Yanhua C., Ip M. Y. K., Smith G. D., Wong T. K. S., Deary I. J. (2013). The structure of stress: confirmatory factor analysis of a Chinese version of the stressors in Nursing Students Scale (SINS). *Nurse Education Today*.

[37] de Souza A. C., Alexandre N. M. C., de Brito Guirardello E., de Souza A. C., Alexandre N. M. C., de Brito Guirardello E. (2017). Psychometric properties in instruments evaluation of reliability and validity. *Epidemiologia e Serviços de Saúde*.

[38] Polit D. F., Beck C. T. (2006). The content validity index: are you sure you know what's being reported? Critique and recommendations. *Research in Nursing & Health*.

[39] Shi J., Mo X., Sun Z. (2012). Content validity index in scale development. *Zhong Nan Da Xue Xue Bao Yi Xue Ban*.

[40] Guo L., Jones M. C., Liu Y., Yv S., Zhu Y., Guo Y. (2019). Cross-cultural validation of the Student Nurse Stress Index Scale: a descriptive survey targeting student nurses in China. *Journal of Affective Disorders*.

[41] Chen X., Velez J. C., Barbosa C. (2014). Smoking and perceived stress in relation to short salivary telomere length among caregivers of children with disabilities. *Stress*.

[42] Baker M. (2012). *Nursing student stress and demographic factors*.

[43] Shukla A., Kalra G. A., Pakhare A. (2013). Understanding stress and coping mechanisms in Indian student nurses. *Sri Lanka Journal of Psychiatry*.

[44] Sarikoc G., Bayram Demiralp M., Oksuz E., Pazar B. (2017). Turkish version of the student nurse stress index: validity and reliability. *Asian Nursing Research*.

[45] Watson R., Deary I., Thompson D., Li G. (2008). A study of stress and burnout in nursing students in Hong Kong: a questionnaire survey. *International Journal of Nursing Studies*.

[46] Sun L., Gao Y., Yang J., Zang X. Y., Wang Y. G. (2016). The impact of professional identity on role stress in nursing students: a cross-sectional study. *International Journal of Nursing Studies*.

[47] Smith G. D., Yang F. (2017). Stress, resilience and psychological well-being in Chinese undergraduate nursing students. *Nurse Education Today*.

[48] Van Hoek G., Portzky M., Franck E. (2019). The influence of socio-demographic factors, resilience and stress reducing activities on academic outcomes of undergraduate nursing students: a cross-sectional research study. *Nurse Education Today*.

[49] Zhou H., Liu M., Zeng J., Zhu J. C. (2016). Selection of nursing teaching strategies in mainland China: a questionnaire survey. *Nurse Education Today*.

[50] Guo L., Söderhamn U., McCallum J. (2017). Testing and comparing two self-care-related instruments among older Chinese adults. *PLoS One*.

[51] Zou G. Y. (2012). Sample size formulas for estimating intraclass correlation coefficients with precision and assurance. *Statistics in Medicine*.

[52] Landis J. R., Koch G. G. (1977). The measurement of observer agreement for categorical data. *Biometrics*.

[53] Burke S., Miller E., Bakas T., Cooper D. (2019). Content validity of the developmental care scale for neonates with CHD. *Cardiology in the Young*.

[54] Zauszniewski J. A., Bekhet A. K. (2012). Screening measure for early detection of depressive symptoms: the depressive cognition scale. *Western Journal of Nursing Research*.

